# Early Postoperative Weight-Bearing Ability after Total Hip Arthroplasty versus Bipolar Hemiarthroplasty in Elderly Patients with Femoral Neck Fracture

**DOI:** 10.3390/jcm13113128

**Published:** 2024-05-27

**Authors:** Chiara Grabmann, Ibrahim Hussain, Anne Zeller, Sertac Kirnaz, Vincent Sullivan, Fabian Sommer

**Affiliations:** 1 University Hospital of Munich, Ludwig Maximilian University, 81377 Munich, Germany; 2 New York Presbyterian Hospital, Weill Cornell Medicine, New York, NY 10065, USA; 3 Department of Biological Sciences, University of South Carolina, Columbia, SC 29208, USA

**Keywords:** fracture, femoral neck, hip, arthroplasty, hemiarthroplasty, weight bearing, mobilization

## Abstract

**Background:** Femoral neck fractures are among the most common types of fractures and particularly affect elderly patients. Two of the most common treatment strategies are total hip arthroplasty (THA) and bipolar hemiarthroplasty (BA). However, the role of the different treatment strategies in the postoperative weight-bearing ability in the early postoperative phase is still not entirely clear. **Methods:** Patients who underwent either THA or BA were consecutively included in our prospective cohort study. Gait analysis was performed during the early postoperative period. The gait analysis consisted of a walking distance of 40 m coupled with the turning movement in between. During the gait analysis, the duration of the measurement, the maximum peak force and the average peak force were recorded. **Results:** A total of 39 patients were included, 25 of whom underwent BA and 14 of whom underwent THA. The maximum peak force during the gait analysis was, on average, 80.6% ± 19.5 of the body weight in the BA group and 78.9% ± 21.6 in the THA group. The additionally determined average peak force during the entire gait analysis was 66.8% ± 15.8 of the body weight in the BA group and 60.5% ± 15.6 in the THA group. **Conclusions:** Patients with femoral neck fractures undergoing THA and BA can achieve sufficient weight bearing on the operated leg in the early postoperative period. In our study, BA did not allow for a significantly higher average and maximum loading capacity compared with THA.

## 1. Introduction

Hip fractures, especially the subgroup of femoral neck fractures, are among the most common types of fractures and particularly affect elderly patients [1]. This is a global health problem that is particularly prevalent in older women [2]. The region of the proximal femur is anatomically exposed and usually has a relatively low amount of protective soft tissue coverage, which increases the injury risk in case of a fall on the hip [3,4]. Contrarily, young people generally have better bone stability and femoral neck fractures rarely occur and are in most cases a consequence of high energy trauma, such as traffic accidents [5,6]. In elderly people, bone stability physiologically decreases with age, and the natural degradation of bones combined with osteoporosis can have a significant impact on bone quality [1]. Additionally, the soft tissue covering of the hip region decreases in elderly people, affording less protection against impacts from falling [4,7]. Therefore, in elderly patients, low impact trauma, such as a light fall on the hip, can lead to femoral neck fractures [8]. In addition to the cause of the injury, the treatment goals also differ between these two age groups. While younger people usually have a higher functional demand and can undergo more complex surgical procedures and post-treatment regimens, older people are often functionally limited and have more comorbidities that increase the risk of surgery [1,9,10]. Taking this into consideration, the treatment goal in geriatric patients is early mobilization with ideally postoperative full weight bearing to reduce the risk of postoperative complications due to immobilization [11,12]. Prolonged immobilization has been shown to increase both mortality and the risk of irreversible loss of mobility [13,14]. Thus, the earliest possible postoperative mobilization is one of the main treatment goals in elderly patients [11,12,15]. For this reason, surgical techniques that require postoperative weight-bearing restrictions and do not permit the patient to apply full weight bearing postoperatively should be avoided. For the evaluation of weight-bearing capacity, special devices have been available recently, which are not larger than a normal insole and can be inserted into the patient’s shoes to evaluate the individual weight-bearing capacity of the affected leg. These devices have already been used in other studies for the scientific evaluation of postoperative weight-bearing capacity and demonstrated a simple applicability and relatively precise measurement results [16].

**Figure 1:**
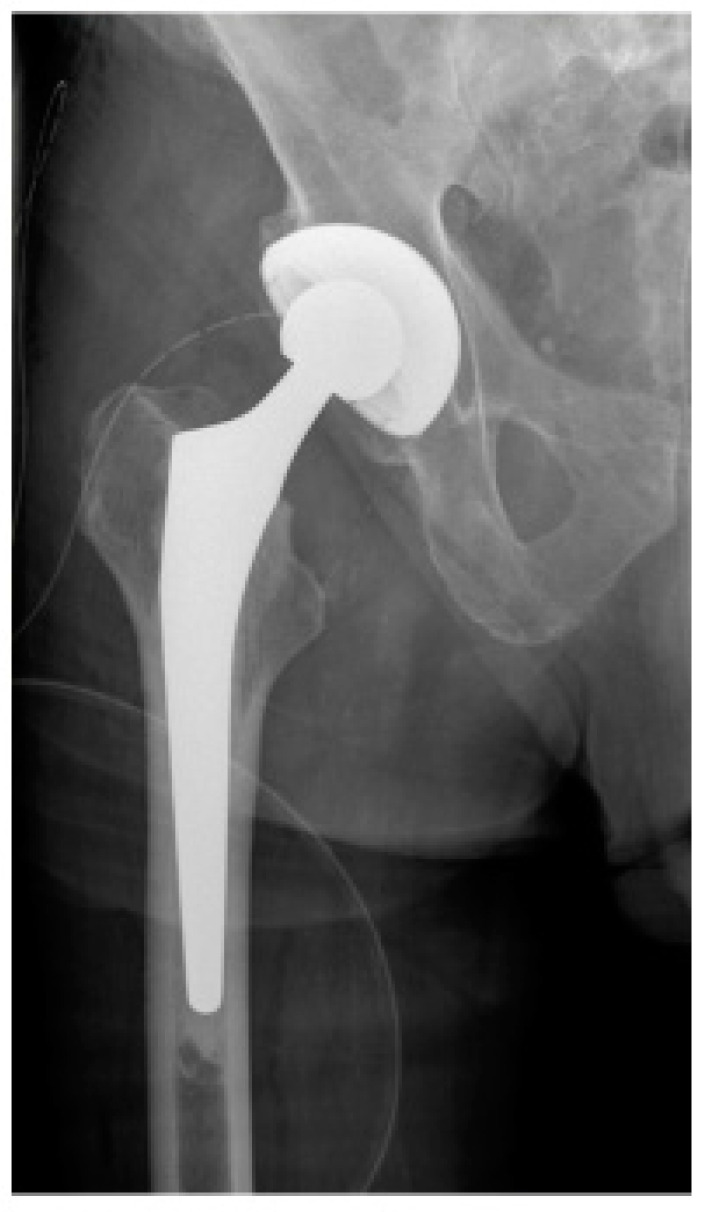
Early postoperative X-ray of the right hip joint in AP view of a male patient after femoral neck fracture who underwent total hip arthroplasty (THA).

**Figure 2:**
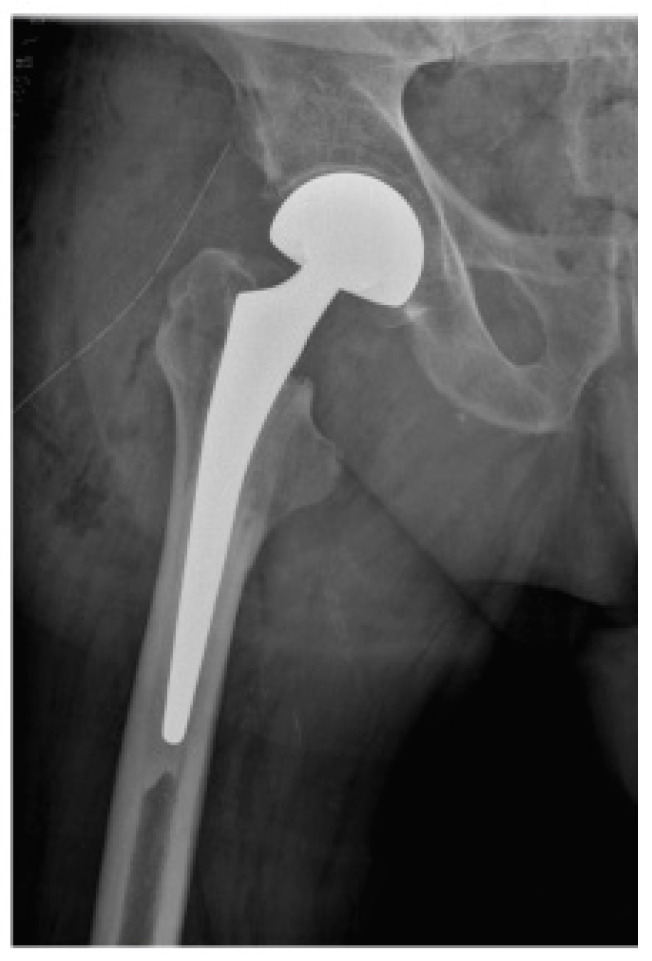
Early postoperative X-ray of the right hip joint in AP view of a male patient after femoral neck fracture who underwent bipolar hemiarthroplasty (BA).

Early mobilization has a significant impact on the further course of rehabilitation and the final functional outcome for the patient [11,15]. To meet this therapeutic goal, the first treatment option for femoral neck fractures in geriatric patients in many cases is direct joint replacement [17,18]. Two of the most common procedures are total hip arthroplasty (THA) (Figure 1), which consists of a replacement of both the femoral head and acetabulum, and bipolar hemiarthroplasty (BA) (Figure 2), in which only the femoral head is replaced [17,19].

BA is typically the shorter and less invasive surgery, since the procedure steps to prepare the acetabulum for an implant are not necessary [20,21]. On the other hand, THA could show a better outcome in patients with pre-existing coxarthrosis and a higher functional demand [17,22,23,24]. However, the role of the different treatment strategies in the postoperative weight-bearing ability in the early postoperative phase is still not entirely clear. In patients without a clear indication for either surgical procedure, the postoperative weight-bearing ability, and thus, the possibility of early mobilization, may be a crucial factor to consider when choosing the best surgical treatment. Furthermore, an evaluation of the postoperative weight-bearing ability could also help patients who have a clear indication for either procedure to more accurately adjust postoperative mobilization regimens and pain medication to achieve the best possible functional outcome.

For this reason, our group evaluated and compared the weight-bearing ability in the early postoperative period in elderly patients with femoral neck fracture who underwent THA or BA as part of a prospective cohort study.

## 2. Materials and Methods

To evaluate the early postoperative weight-bearing ability in elderly patients after hip replacement surgery, we conducted a prospective cohort study. The study was reviewed and approved by the Institutional Review Board (IRB) (Ethic Committee Name: Ethic Committee of the Ludwig Maximilian University of Munich (LMU), Approval Code: 214-16, Approval Date: 28 June 2016). Patients over the age of 60 years who underwent surgical treatment for a femoral neck fracture, either THA or BA, were consecutively included in the study. Our study focused exclusively on fractures in the femoral neck region that can be treated with THA or BA, following the recommendations of the AO Foundation. These include dislocated subcapital, transcervical and basicervical femoral neck fractures, according to AO classifications 31B1, 31B2 and 31B3 [25]. Patients who were not able to perform a postoperative gait analysis were excluded. These included patients with relevant cognitive impairment (for example, dementia, postoperative delirium), pre-existing immobility (for example, bedridden patients, musculoskeletal disorders) and other restricting comorbidities that were limiting postoperative mobilization.

Prior to the gait analysis, cognitive impairment, the presence of comorbidities and the mobility of patients before trauma were assessed using standardized questionnaires. The Mini-Mental State Examination was used to evaluate cognitive impairment [26]. Comorbidities were assessed using the Charlson Index [27]. Patients’ mobility and daily living ability were assessed using the Parker Mobility Score (PMS) and the Barthel Index (BI) [28,29]. These functional scores were assessed for the first time right after hospital admission to estimate the functional scores before the accident at the earliest possible time point and for the second time on the date of the postoperative gait analysis.

Postoperatively, all patients were treated according to the World Health Organization (WHO) standardized pain management guidelines [30]. During the whole study, all patients were mobilized by the same experienced physiotherapy team. All patients were allowed full weight bearing immediately following operation without weight-bearing restrictions, meaning the operated leg was allowed to be loaded with the full body weight. Prior to mobilization, patients were provided with an appropriate dose of analgesia, according to the WHO pain management guidelines, depending on their individual pain level, if necessary, in order to facilitate mobilization with minimum pain.

**Figure 3:**
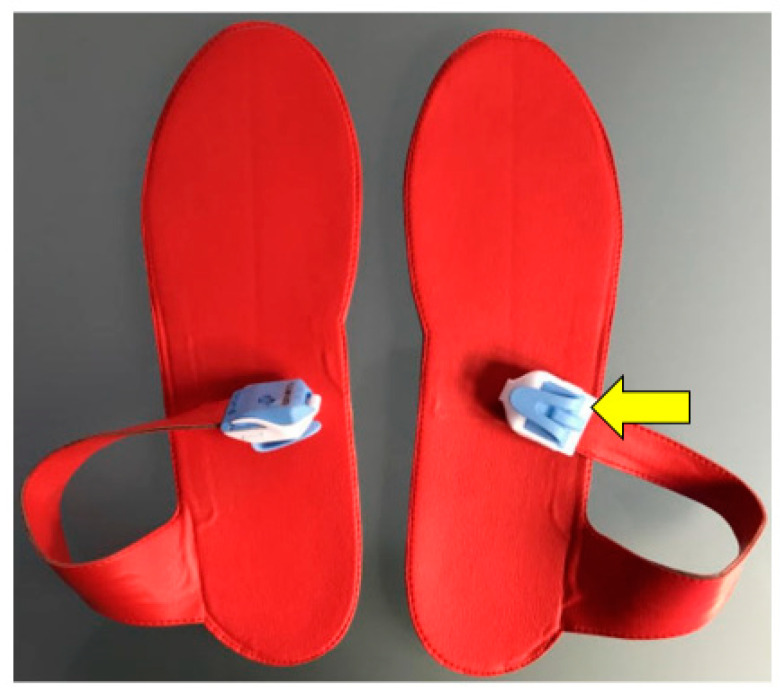
Example of a pair of insoles with integrated force sensors that were used for the gait analysis. The electronic hardware is located in a small box (yellow arrow) that is connected to the soles by a cable. The insoles are about 2–3 mm thick, so the soles do not significantly interfere with walking.

**Figure 4:**
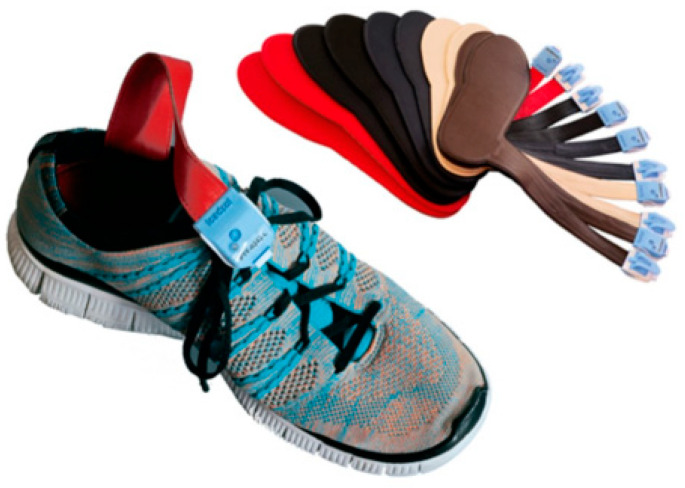
Examples of insoles with different sizes that were used for the gait analysis, each size being assigned a different color. The insoles were selected individually to match the patients’ shoe size and placed in the patients’ shoes before the gait analysis. The small box containing the hardware is attached to the outer shoe, where it does not interfere with walking.

Postoperative gait analysis was performed using insoles with integrated force sensors (Figure 3). The insoles were matched to the patients’ shoe size and placed in both shoes before the gait analysis. The insoles’ force sensor measures the actual applied plantar force between the foot and the shoe and reflects the load on the leg with each step. The measuring range of the pressure sensors in the model used ranges from 20 N to 2500 N at a maximum sampling rate of 200 Hz, meaning 200 measurements per second. The electronic hardware for recording and transmitting was located in a small box connected to the soles by a cable and was attached to the outer shoe during the test (Figure 4). The weight of the box was only a few grams and did not cause the patients any impairment during the gait analysis. The data were transmitted via Bluetooth to a mobile tablet PC during the gait analysis and stored using a dedicated software application (loadsol version 1.4.72). Before each measurement, the sensor soles were calibrated. All measurements in the study were performed with the same test setup.

Gait analysis was performed during the hospital stay in the early postoperative period and was adapted to the individual patient’s mobilization ability. During the gait analysis, the duration of the measurement in seconds [s], the maximum peak force and the average peak force in Newton [N] were recorded by the insoles. The maximum peak force is defined as the highest load that the patient is able to apply during the measurement period and usually occurs during walking in the phase when the foot hits the ground. This value depends on the walking speed and can exceed 100% of the body weight due to the kinetic energy acting in addition to the body weight. The average peak force is a parameter calculated by the software application that takes the measurements of the applied force during each step and calculates an average value for all steps during the measurement period. To avoid bias from inconsistent force application, at the beginning of walking, the first three steps from each recording period were excluded from the determination of the average peak force. In addition to the load, the walking speed in meters per second [m/s] was calculated. These parameters were determined for all patients in the BA group and the THA group and compared to determine whether one of the two treatment groups was able to show a higher postoperative weight-bearing capacity. Walking speed was measured to estimate the impact of the additional kinetic energy generated during walking on our measurements.

For the gait analysis, patients were provided with a walking aid of their choice (crutches, walker or rollator). The decision regarding which type of walking aid was used depended on the individual patient’s ability to bear weight and coordination. Crutches were used for patients with good coordination, and a walker or rollator was used for patients with impaired coordination. The walking aid was initially used in all patients and was then adjusted and reduced in further course based on the patient’s individual load-bearing capacity and individual coordination ability. In our study, the walking aid served to reduce the load on the extremity and to prevent falls during mobilization. For this reason, the walking aid continued to be used in some patients who were already able to bear weight sufficiently but had an increased risk of falling due to impaired coordination. Because the use of the walker was not exclusively linked to weight-bearing capacity, this parameter was not included in our analysis due to its limited informative value. During mobilization, all patients were supported by a physiotherapist who ensured correct movement and was present for fall prevention. The sequence of the load measurement was the same for all patients and consisted of a walking distance of two 20 m stretches with a 180 degree turn in between. In total, the gait analysis consisted of a walking distance of 40 m coupled with the turning movement in between.

### Statistical Analysis

A statistical analysis of the results was performed using the IBM SPSS software (Version 28.0, IBM corporation, Armonk, NY, USA). Before comparing the two groups, the parameters were tested for normal distribution using the Shapiro–Wilk test. For the comparison of maximum peak force and average peak force during walking, the unpaired *t*-test was used to identify significant differences between the two groups. Due to lack of normal distribution in the score values, the non-parametric Mann–Whitney-U-test was used for comparing the score values between the two groups. The significance level was set at *p* < 0.05.

## 3. Results

A total of 39 patients with femoral neck fracture met the inclusion criteria and were included in the study. Depending on the discretion of the operating surgeon, who was not directly involved in this study, 25 (14 male/11 female) patients underwent BA, and 14 (6 male/8 female) patients underwent THA. The mean age in the BA group was 82.5 ± 7.0 years and 74.0 ± 7.9 years in the THA group (Table 1). Age was normally distributed in both groups and presented no statistically significant difference between groups.

### 3.1. Postoperative Weight Bearing

**Figure 5:**
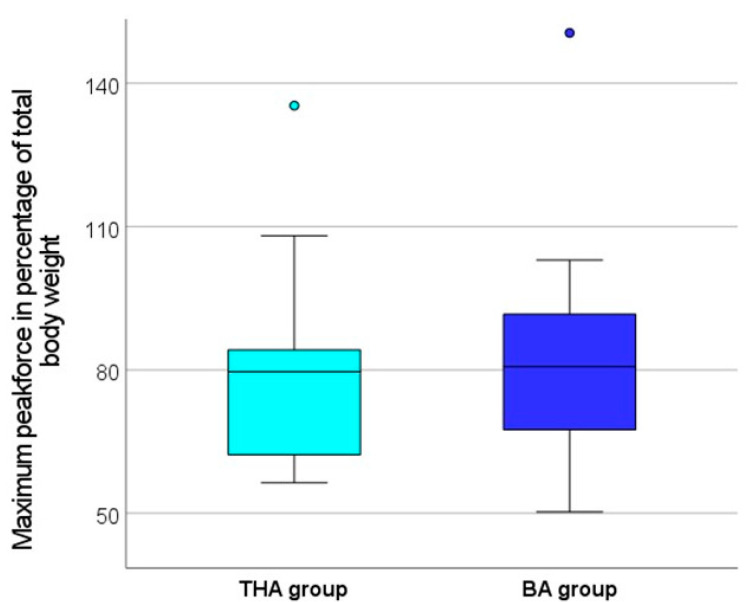
Box plot comparing the maximum peak force during the gait analysis between the BA group and the THA group. The maximum peak force is presented in relation to the body weight of the study participants. There was no significant difference (*p* = 0.799) between both groups.

**Figure 6:**
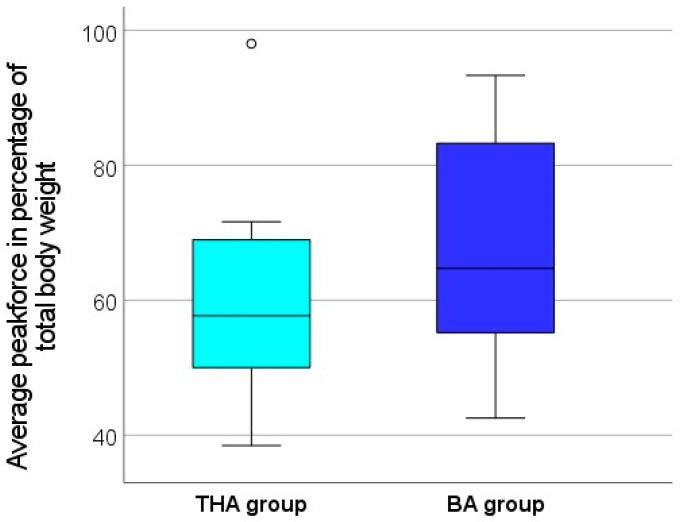
Box plot comparing the average peak force during the gait analysis between the BA group and the THA group. The average peak force is presented in relation to the body weight of the study participants. There was no significant difference (*p* = 0.272) between both groups.

The evaluation of the gait analysis showed that both groups were able to achieve a maximum load of more than 75% of their own body weight, on average, during walking. The maximum peak force during the gait analysis was, on average, 80.6% ± 19.5 of the body weight in the BA group and 78.9% ± 21.6 in the THA group (Figure 5). The difference between the two groups was not statistically significant (*p* = 0.799). The additionally determined average peak force (average of maximum force for each step) during the entire gait analysis was 66.8% ± 15.8 of the body weight in the BA group and 60.5% ± 15.6 in the THA group (Figure 6). Although the average peak force was found to be around 6 points higher in the BA group, the difference between the two groups was not statistically significant (*p* = 0.272). Due to a connection loss between the soles and the mobile tablet PC during the recording of the average peak force, this value was not saved for two patients from the THA group and for four patients from the BA group; therefore, they could not be included in the evaluation. This referred only to the average peak force; all other parameters were transferred correctly by the software.

Walking speed was, on average, 0.31 m/s ± 0.14 in the BA group and 0.32 m/s ± 0.19 in the THA group. Thus, no significant difference was found in walking speed between the two patient groups (*p* = 0.890). The difference in walking speed between the two groups was not statistically significant.

### 3.2. Mobility and Comorbidity

A comparison of the patients’ mobility and health-related quality of life before trauma and after surgery, as assessed by the PMS and BI, revealed no significant difference between the two groups.

## 4. Discussion

Our study showed no significant difference in postoperative weight bearing in the early postoperative period in patients with femoral neck fracture, regardless of whether they underwent THA or BA. Both groups were able to achieve a maximum peak force of more than 75% of their own body weight, on average, on the operated leg during postoperative mobilization. A healthy person can statically load their leg with 100% of their body weight while standing. In the case of a dynamic load, such as walking or running, this value can also increase to more than 100% of the body weight, since, in addition to the body weight, the kinetic energy also puts force on the limb. In our study, however, postoperative mobilization was performed very slowly in all cases, since the physiotherapy team primarily focused on the correct execution of the movement sequence and not on the speed. Therefore, very low kinetic energies occurred when the leg touched the ground. In order to better quantify the influence of kinetic energy on our measurements, walking speed was additionally measured. In healthy subjects, the walking speed is about 1.5 m/s [31]. In our study, the walking speed in both groups was about 0.3 m/s, which is only about 20% of the normal walking speed. Since walking speed affects kinetic energy mathematically to the second power, a reduction in speed to 20% lowers the total corresponding kinetic energy to 4%. Due to this particular feature in the mobilization, the additional kinetic energy acting on the limb during walking is considered low and can be practically neglected for better comparability. This is also evident in the observation that the maximum peak load in our measurements, which usually occurs when the foot hits the ground, is above 100% in only a few cases (see Figure 5).

There is a consensus in the guidelines of several countries that the earliest possible postoperative mobilization should be a treatment goal in elderly trauma patients [32,33,34,35]. This treatment strategy showed a reduced complication rate and improved long-term outcome [11,12,15]. For these reasons, BA is often preferred in elderly patients with femoral neck fracture.

The choice of the surgical procedure depends on several additional factors, such as age, functional expectations, the amount of displacement of the fracture fragments or the patient’s comorbidities [19,36]. BA typically represents the less invasive procedure of the two treatment strategies [20,21]. The average duration of surgery of a BA is shorter, and it has less average blood loss compared to a THA [20,21]. However, one of the disadvantages of BA is the high rate of painful acetabular erosions in the long term. In the literature, the rate of acetabular erosions in BA is described as being up to 36% [24,37]. This is another reason why the revision rate of BA within the first few years is higher compared to THA [22]. Therefore, the indication for BA is mainly given in elderly patients with a low functional demand and an increased operative risk. Even in this group, due to the growing life expectancy over the last decades, these implants may lead to complaints over the years [38].

Both surgical procedures have different advantages and disadvantages and their corresponding indications. Mobile and independent elderly patients with femoral neck fractures usually benefit more from THA. In the long term, THA has a better functional outcome and has fewer revisions than hemiarthroplasty [22,23,24]. A major disadvantage of the procedure is a higher risk of dislocation, particularly in the first period after surgery [22,23]. In addition, the operation takes longer and usually causes greater blood loss during the surgery, since the acetabular bone needs to be prepared for the hip cup implant [20,21]. Overall, THA represents the more invasive surgical procedure but is associated with better joint function in the long term than BA.

Which of the procedures, BA or THA, offers advantage for the actual postoperative weight-bearing ability of geriatric patients has not yet been investigated, and it is an important factor to consider when choosing the right treatment for a patient. Another study has already shown that hip replacement surgery allows effective loading in the early postoperative period [16]. However, the relatively small sample size in the described study specifically limits the findings related to the THA group. The extent of how much the postoperative weight bearing differs in detail between BA and THA could only be assessed to a limited extent due to the small number of cases, consisting of fewer than 10 cases in the THA group in the mentioned study.

There are clear indications for both surgical procedures where one procedure is more suitable and the patient is more likely to benefit from one particular procedure. In between, however, there are also patients without clear indications for one of the two procedures and who are suitable for both operative techniques. Patients without a clear indication for BA or THA are particularly challenging for the decision-making surgeon. Especially in older patients, where early postoperative mobilization is the therapeutic goal, Bhandari et al. were able to show that surgeons tend to prefer BA in case of doubt [18]. In this regard, some patients without a clear indication for either procedure could potentially benefit from the higher functionality of THA.

Our analysis showed no significant differences in weight bearing after either surgical procedure in the early postoperative period. Patients treated with both surgical techniques, THA and BA, were able to bear sufficient weight on the operated limb postoperatively. BA did not show a significant advantage for older patients to allow earlier full weight bearing compared to patients who underwent THA. This finding should be taken into consideration when planning the surgical treatment strategy.

Nevertheless, our study has a few limitations. It must be taken into account that the measurement took place at an early postoperative point of time and thus does not provide any information on the load-bearing ability in the long-term course. However, mobility in the early postoperative period during the hospital stay represents an important prognostic factor for the outcome of the patients [39]. Early mobilization, within 48 h of surgery, showed an increased functional recovery. Additionally, a higher rate of early mobilized patients were able to return directly back home after the surgery and showed lower rates of discharge to high-level care centers [39]. Several international guidelines recommend starting the postoperative mobilization on the first postoperative day for geriatric patients with femoral neck fracture if no contraindications exist [33,34]. Patients with femoral neck fracture lose, on average, more than 50% of their muscle strength within the first postoperative week in the affected leg compared to the non-affected leg, which affects the mobilization of elderly patients in particular [40,41,42,43]. For these reasons, we decided to conduct our evaluation in the early postoperative period.

Another limiting factor of our study is the sample size. In our study, only patients who were able to perform the gait analysis correctly were included, since this was the only way to obtain valid and comparable measurements. This resulted in a smaller sample size, but the group size of our study is comparable with other gait analysis studies that have used similar measurement procedures with sensor soles in determining the postoperative weight-bearing ability [44,45]. Due to the selection beforehand, all included patients were able to successfully perform gait analysis under appropriate guidance, and no patient needed to be excluded due to inability to follow the instructions during the gait analysis.

In addition, it is theoretically possible that our exclusion criteria might contribute to a selection bias. Since we did not include bedridden and severely mentally impaired patients in our analysis, we cannot draw any conclusions regarding this group of patients. For bedridden patients, this might be of limited significance, since weight bearing was already impossible before the injury. However, it could be that postoperative weight bearing differs between the two procedures in cognitively impaired patients and that this was not noticed due to the exclusion of this patient group. However, severely mentally impaired patients would not primarily be considered as ideal candidates for THA because of the higher postoperative dislocation and complication risk; therefore, this potential bias might be primarily of theoretical interest. Furthermore, the exclusion was made because we do not currently have a valid method to obtain reproducible and comparable data from patients who do not adhere exactly to the procedural instructions. As soon as we have the possibility to do so, we will include this patient group in future weight-bearing studies. In order to avoid selection bias and increase comparability, we limited our evaluation exclusively to patients with traumatic hip fractures and excluded patients who underwent elective THA due to advanced osteoarthritis of the hip joint, since mobilization of patients after traumatic injuries is typically more challenging than after elective surgery.

The second potential bias in our evaluation is a recall bias, since functional scores before the accident were determined based on the patient’s memory. This can result in the subjectively perceived mobility before the accident being classified as too high or too low. For elective surgery, it is possible to obtain the functional scores directly before the operation or even to determine them directly. This is not possible in the case of accidents, since it is impossible to anticipate an accident, and therefore, it is not possible to collect data in advance. However, since our study only evaluates patients after an accident, this is the most accurate way to determine mobility before the accident. In addition, to minimize the risk of recall bias, patients were asked about their pre-accident mobility as soon as possible after hospital admission. In addition, we focused the evaluation on the objective data from our weight-bearing analysis and not on the subjective information provided by the patients.

The focus of our study was to determine and compare the actual weight-bearing ability of older patients with femoral neck fracture in the early postoperative period who underwent either THA or BA. Our study showed no significant difference between the two procedures regarding the weight-bearing ability. This might indicate that the effect of the comparatively more invasive procedure of THA on the postoperative weight-bearing ability of older patients may be overestimated. For a more accurate evaluation, future studies should investigate weight bearing after THA and BA in the medium- and long-term course and with an increased sample size.

## 5. Conclusions

Our study shows that older patients with femoral neck fracture undergoing THA and BA can achieve sufficient weight bearing on the operated leg in the early postoperative period. In our study, BA did not allow for a significantly higher average and maximum loading capacity in the early postoperative period compared with THA, meaning there were no relevant differences in terms of weight-bearing ability between the two groups. In our study, we were able to quantify the difference and objectify this hypothesis. This finding should be considered during the decision-making process of surgical treatment, especially in patients without a clear indication for one of the two surgical procedures. The extent to which the weight-bearing ability differs between the two surgical procedures in the long term must be shown in further studies.

## Figures and Tables

**Table 1:** Demographics of patients in the THA group and BA group.

	THA	BA
Number in total	14	25
Age, mean ± SD	74.0 ± 7.9 years	82.5 ± 6.9 years
Sex		
Male	6 (43%)	14 (56%)
Female	8 (57%)	11 (44%)
ASA, mean ± SD	2.6 ± 0.6	2.9 ± 0.5

ASA: American Society of Anesthesiologists physical status classification system.

## Data Availability

Dataset available on request from the authors.
